# Tuberculosis Therapy Modifies the Cytokine Profile, Maturation State, and Expression of Inhibitory Molecules on *Mycobacterium tuberculosis*-Specific CD4^+^ T-Cells

**DOI:** 10.1371/journal.pone.0158262

**Published:** 2016-07-01

**Authors:** Kapil K. Saharia, Constantinos Petrovas, Sara Ferrando-Martinez, Manuel Leal, Rafael Luque, Prudence Ive, Anne Luetkemeyer, Diane Havlir, Richard A. Koup

**Affiliations:** 1 Institute of Human Virology and Division of Infectious Diseases, University of Maryland School of Medicine, Baltimore, MD, United States of America; 2 Immunology Laboratory, Vaccine Research Center, National Institute of Allergy and Infectious Diseases, National Institutes of Health, Bethesda, MD, United States of America; 3 Laboratorio de InmunoBiología Molecular, Hospital General Universitario Gregorio Marañón, Madrid, Spain, Instituto de Investigación Sanitaria Gregorio Marañón, Madrid, Spain; 4 Laboratory of Immunovirology, Clinic Unit of Infectious Diseases, Microbiology and Preventive Medicine, Institute of Biomedicine of Seville, IBiS, Virgen del Rocío University Hospital, Seville, Spain; 5 Department of Infectious Diseases, Microbiology and Preventive Medicine, Virgen del Rocío University Hospital, Seville, Spain; 6 Clinical HIV Research Unit, Department of Internal Medicine, Faculty of Health Sciences, University of the Witwatersrand, Johannesburg, South Africa; 7 Division of HIV, Infectious Diseases and Global Medicine, Zuckerberg San Francisco General Hospital, University of California San Francisco, San Francisco, CA, United States of America; IPBS, FRANCE

## Abstract

**Background:**

Little is known about the expression of inhibitory molecules cytotoxic T-lymphocyte antigen-4 (CTLA-4) and programmed-death-1 (PD-1) on *Mycobacterium tuberculosis* (Mtb)-specific CD4 T-cells and how their expression is impacted by TB treatment.

**Methods:**

Cryopreserved PBMCs from HIV-TB co-infected and TB mono-infected patients with untreated and treated tuberculosis (TB) disease were stimulated for six hours with PPD and stained. Using polychromatic flow cytometry, we characterized the differentiation state, cytokine profile, and inhibitory molecule expression on PPD-specific CD4 T-cells.

**Results:**

In our HIV-TB co-infected cohort, TB treatment increased the proportion of PPD-specific CD4 T-cells co-producing IFN-γ^+^IL-2^+^TNF-α^+^ and IFN-γ^+^IL-2^+^ (p = 0.0004 and p = 0.0002, respectively) while decreasing the proportion of PPD-specific CD4 T-cells co-producing IFN-γ^+^MIP1-β^+^TNF-α^+^ and IFN-γ^+^MIP1-β^+^. The proportion of PPD-specific CD4 T-cells expressing an effector memory phenotype decreased (63.6% vs 51.6%, p = 0.0015) while the proportion expressing a central memory phenotype increased (7.8% vs. 21.7%, p = 0.001) following TB treatment. TB treatment reduced the proportion of PPD-specific CD4 T-cells expressing CTLA-4 (72.4% vs. 44.3%, p = 0.0005) and PD-1 (34.5% vs. 29.2%, p = 0.03). Similar trends were noted in our TB mono-infected cohort.

**Conclusion:**

TB treatment alters the functional profile of Mtb-specific CD4 T-cells reflecting shifts towards a less differentiated maturational profile and decreases PD-1 and CTLA-4 expression. These could serve as markers of reduced mycobacterial burden. Further study is warranted.

## Introduction

*Mycobacterium tuberculosis* (Mtb) infection remains a leading cause of morbidity and mortality worldwide. However, the vast majority of infected individuals never develop clinical disease as the host immune response controls the organism. While the precise mechanisms leading to loss of immune-control and disease reactivation remain elusive, it is well established that CD4 T-cells are critical in controlling tuberculosis (TB) infection [1–5]. CD4 T-cells not only maintain the integrity of granulomas in TB infection [6, 7], but are a major source of interferon gamma (IFN-γ), which can promote macrophage killing of *M*. *tuberculosis* through induction of autophagy or through increased expression of antimicrobial peptides [8, 9].

The ability of antigen-specific T-cells to produce simultaneously multiple cytokines has been associated with control of chronic viral infections [10–12]. Recently, several groups have identified distinct cytokine signatures in patients with active and latent TB infection [13–16]. The prevailing thought is that states of lower mycobacterial burden are characterized by a higher frequency of polyfunctional T cells, although not all groups agree.

Progressive T-cell dysfunction, characterized by reduced proliferative capacity and effector functions, has been described in the setting of chronic viral infections [17]. This T-cell dysfunction is mediated by several inhibitory pathways including the programmed death-1 (PD-1) and cytotoxic T-lymphocyte antigen 4 (CTLA-4) pathways. Both PD-1 and CTLA-4 are upregulated on activated T-cells and binding of the T-cell receptor to either of these molecules decreases T-cell activation and promotes cell cycle arrest [18]. PD-1 expression on HIV-specific CD4 and CD8 T-cells and CTLA-4 expression on HIV-specific CD4 T-cells is directly correlated with HIV viremia [19–21]. Increased PD-1 and CTLA-4 expression on HCV-specific CD8 T-cells within the liver has been observed in chronic HCV infection [22, 23]. In active TB disease, PD-1 is upregulated on CD3 T-cells and blockade of the PD-1 pathway enhances IFN-γ production [24]. However, little is known about the expression of these molecules on Mtb-specific CD4 T-cells.

We set out to explore the relationship between PD-1 and CTLA-4 expression on Mtb-specific CD4 T-cells and mycobacterial burden by studying patients undergoing treatment for TB disease. We show that reductions in mycobacterial burden through TB treatment result in distinct changes in the cytokine profile of Mtb-specific CD4 T-cells and these changes reflect shifts in the maturation state of Mtb-specific CD4 T-cells but may also be related to diminished expression of PD-1 and CTLA-4.

## Materials and Methods

This study was reviewed and approved by the Institutional Review Board of the National Institute of Allergy and Infectious Diseases (NIAID), National Institutes of Health (NIH). The use of specimens from AIDS Clinical Trial Group (ACTG) 5221 was approved by the appropriate ACTG Review Committee. All ACTG 5221 study volunteers provided written informed consent to store samples for future use. Study volunteers from the Tuberculosis Clinic of the Hospital Universitario Virgen del Rocío, Seville, Spain also provided written informed consent for use of samples. Use of these samples was approved by the ethics committees at NIAID, NIH and Universitario Virgen del Rocío, respectively.

### Study Subjects

AIDS Clinical Trial Group 5221 (A5221) was an open-label, randomized trial comparing earlier anti-retroviral therapy (ART) to later ART in persons with HIV-1 infection and CD4 T-cell count less than 250/mm^3^ that were initiating treatment for confirmed or probable tuberculosis disease [25]. Eligibility criteria for A5221 have been previously published [25]. Earlier ART was administered within 2 weeks of and later ART between 8 and 12 weeks after the start of TB treatment. Tuberculosis therapy was provided to patients by the study sites in accordance with the country’s national TB treatment guidelines. Longitudinal cryopreserved peripheral blood mononuclear cell (PBMC) samples were collected from a subset of individuals enrolled in A5221. We included in our study subjects from A5221 with sufficient stored PBMC from baseline (within one month of starting TB therapy) and week 48 (between 36 and 48 weeks after randomization).

Because co-administration of ART to patients enrolled in A5221 could potentially confound our results, we enrolled a cross-sectional cohort of HIV negative individuals with untreated and treated TB disease. Study participants were recruited from the Tuberculosis Clinic of the Hospital Universitario Virgen del Rocío, Seville, Spain and TB therapy was provided in accordance with national TB treatment guidelines.

### Antibodies

The following directly conjugated antibodies were used: CD3-APCH7, CD27-Alexa Fluor700, MIP1β-PE, CTLA4-APC, TNFα-Cy7PE, IL2-Cy55PCP (BD Biosciences); CD4-Cy55PE, Streptavidin (SA)-quantum dot (QD) 655, Aqua amine reactive dye (Invitrogen); CD45RO-TRPE, 2B4-Cy5PE (Beckman Coulter); IFNγ-eFluor450 (eBioscience); Biotinylated PD-1 (clone BAF1086; R&D Systems); CD8-BV711 (Biolegend). The following antibodies were conjugated in our laboratory: CD8-QD800 and CD57-QD565.

### Intracellular cytokine staining assays and flow cytometric analysis

Cryopreserved PBMCs were thawed, washed, and resuspended in R10 media (RPMI-1640 supplemented with 10% heat inactivated fetal calf serum, 100 U/ml penicillin G, 100 U/ml streptomycin sulfate, and 1.7mm sodium glutamine) containing 50 U/ml Benzonase (Novagen). Cells were rested at 37°C for 1 hour before use. PBMCs at a density of 1–2 x 10^6 cells/ml were either left unstimulated (negative control) or stimulated with PPD 10μg/ml (Statens Serum Institut) to detect PPD-specific CD4 T-cell responses. If sufficient cells remained stimulations using 2μl of undiluted CMV grade 2 antigen (Microbix) per 1ml of cell suspension were performed to detect CMV-specific CD4 T-cell responses. Unfortunately, due to low cell recovery rates, a positive stimulation control using staphylococcus enterotoxin B (SEB) could not be performed routinely on PBMC samples. Stimulations were performed in the presence of 1 μg/ml αCD28/αCD49d (BD Biosciences). PBMCs were stimulated at 37°C for 6 hours with Brefeldin A 10 μg/ml (Sigma Aldrich) added after the first hour.

Following stimulation, PBMCs were washed with PBS and stained with Aqua live/dead dye. Cells were surface stained with a pre-titered mixture of directly conjugated antibodies to CD4, CD8, CD27, CD45RO, CD57, PD-1, and 2B4. Cells were washed and stained with Streptavidin before undergoing permeabilization using the cytofix/cytoperm kit (BD Biosciences). Intracellular staining was performed using a pre-titered mixture of directly conjugated antibodies to CD3, CTLA4, IFN-γ, IL-2, MIP-1β and TNF-α. Cells were then washed and fixed with 1% paraformaldehyde and stored at 4°C until analyzed with a modified flow cytometer (LSRII; BD) within 24 hours of staining. Between 200,000 and 1,000,000 events were collected per sample. Electronic compensation was performed using antibody capture beads stained separately with the identical antibodies used in each test sample.

Analytical gating was conducted using FlowJo version 9.2 (Tree Star). Forward scatter and side scatter properties were used to isolate small lymphocytes and remove cell aggregates. A CD3 versus Aqua plot was used to remove dead cells and a CD3 versus IFN-γ plot was used to isolate CD3 cells. A CD4 versus CD8 gate was then applied to select for CD4 T-cells (S1 Fig).

### Statistical methods

Background cytokine production in the negative control samples was subtracted from each antigen stimulated sample using Pestle (v1.7) and graphical representation of polyfunctional cytokine production was performed using Simplified Presentation of Incredibly Complex Evaluations (SPICE, Version 5.21) provided by M. Roederer (National Institutes of Health, Bethesda, MD). Total memory CD4 T-cells that produced IFN-γ, IL-2, TNF-α or MIP-1β following 6 hour stimulation with PPD or CMV grade 2 antigen were classified as PPD-specific or CMV-specific CD4 T-cells, respectively (S1 Fig). Analysis of differentiation stage and inhibitory molecule expression on antigen-specific CD4 T-cells was performed using samples with greater than two-fold higher cytokine response than negative control samples and at least 50 cytokine-positive cells. Experimental variables were analyzed using the nonparametric Mann-Whitney U test or the Wilcoxon matched-pairs signed rank test. The Wilcoxon matched-pairs signed rank test was used to analyze paired data from the A5221 cohort while the Mann-Whitney U test was used to analyze unpaired data from the cross-sectional, HIV negative cohort. Comparisons between cohorts were analyzed using the Mann-Whitney U test. Correlations were performed using the nonparametric Spearman rank test. P-values <0.05 were considered significant. The GraphPad Prism (Version 6.0d; GraphPad Software) statistical analysis program was used for this analysis.

## Results

Forty individuals enrolled in A5221 had sufficient PBMCs from baseline and week 48 time points and were eligible for this study. Nineteen individuals were excluded due to poor specimen viability leaving 21 individuals with paired samples for analysis. To support findings from the A5221 cohort, we further analyzed 15 HIV negative patients, of whom 6 had untreated TB disease and 9 completed therapy for TB disease.

### Characteristics of the Study Population

Clinical characteristics of our A5221 cohort (hereafter referred to as HIV-TB cohort) are presented in Table 1. Sixteen patients (76.2%) had baseline samples within 2 weeks of TB treatment, while 5 individuals had baseline samples within 4 weeks of TB treatment. Thirteen patients received earlier ART while 8 received later ART. All patients achieved virologic suppression by week 48.

**Table 1:** Characteristics of ACTG 5221 (A5221) Study Population.

Characteristic	A5221 Cohort
	N = 21 (%)
**ACTG Group Assignment**	
Early ART	13 (61.9%)
Deferred ART	8 (38.1%)
**Median CD4 T-cell count/mm**^**3**^ **(Interquartile range)**	
Baseline	131 (35–185)
Week 48	274 (152–344)
**Baseline HIV-1 RNA log**_**10**_ **copies/ml**	
Median	5.38
Interquartile range	5.15–5.88
**HIV-1 RNA <400 copies/ml (Week 48), N (%)**	21 (100%)
**TB diagnosis**^$^	
Confirmed	12 (57.1%)
Probable	9 (42.9%)
**AFB Sputum Smear**	
Positive	10 (47.6%)
Negative	2 (9.5%)
Unknown	9 (42.9%)
**M. tuberculosis culture**	
Positive	7 (33.3%)
Negative	11 (52.4%)
Unknown	3 (14.3%)
Site of Infection^*^	
Lungs	19 (90.5%)
Lymph node	2 (9.5%)
Pleural/pericardial	1 (4.8%)

^$^ Confirmed tuberculosis defined by detection of acid-fast bacilli in sputum smear or lymph node sample or by positive culture for *M*. *tuberculosis* from sputum, lymph node, or other sterile site. Probable tuberculosis required clinician’s assessment that signs and symptoms warranted tuberculosis treatment.

^*^ Patients may have more than one site of infection

Within our cross-sectional, HIV negative cohort (hereafter referred to as TB cohort), all 6 patients with untreated TB disease had TB confirmed by either mycobacterial culture or AFB sputum smear. Patients who completed TB treatment (n = 9) were enrolled a median of 12 months (range 5–30 months) from the end of treatment.

### TB treatment alters the cytokine profile of PPD-specific CD4 T-cells

**Fig 1:**
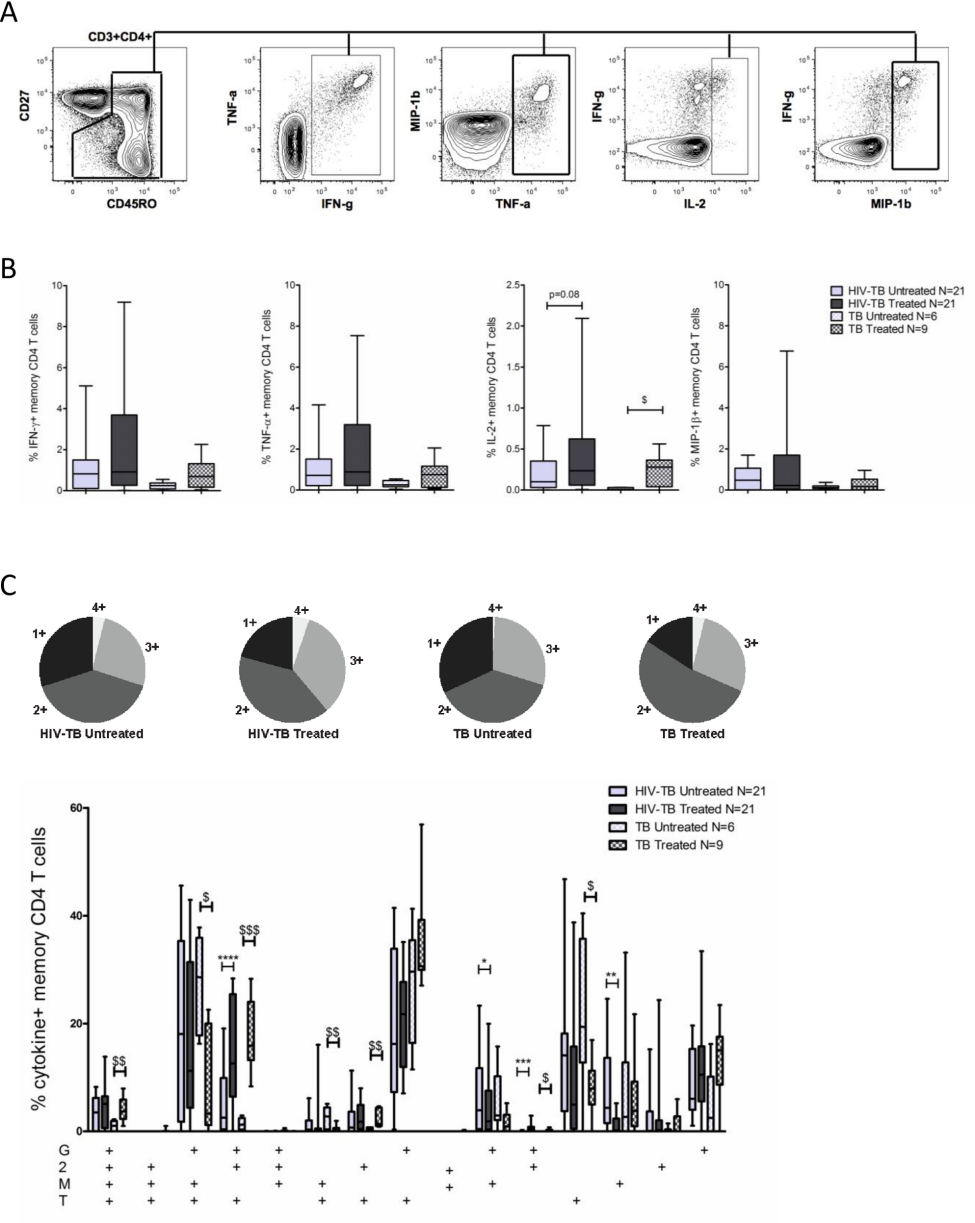
TB therapy alters the functional profile of PPD-specific CD4 T-cells. **A.** Representative plot showing the gating scheme used to identify cytokine/chemokine positive cells. Single, live, CD3^+^, CD4^+^ T cells were gated for CD27 and CD45RO to identify the total CD4 memory population. Cytokine/chemokine gates were then applied to the total CD4 memory population to identify cytokine/chemokine positive cells. **B.** Total frequency of IFN-γ, TNF-α, IL-2, and MIP-1β produced by memory CD4 T-cells in untreated and treated TB disease within our HIV-TB and TB cohorts. Background cytokine/chemokine production from the negative control sample was subtracted. **C.** Pie graph displaying the proportion of cytokine/chemokine^+^ CD4 T-cells producing all 4 cytokines/chemokines (light grey wedge) or any combination of 3 cytokines/chemokines (medium gray), 2 cytokines/chemokines (dark grey), or a single cytokine/chemokine (black wedge) in untreated and treated TB disease. The bar graph depicts the relative contribution of each cytokine/chemokine producing subset to the overall PPD-specific CD4 T-cell response. “G” denotes IFN-γ, “2” denotes IL-2, “T” denotes TNF-α, and “M” denotes MIP-1β. For all bar graphs bars represent the interquartile range (IQR), horizontal lines denote the median, and whiskers the 10^th^ and 90^th^ percentiles. Solid bars represent our HIV-TB cohort, patterned bars represent our TB cohort. Light gray represents untreated TB disease while dark gray represents treated TB disease. Statistical analysis was performed using the Wilcoxon matched-pairs signed rank test for paired data (HIV-TB cohort) and the Mann-Whitney test for unpaired data (TB cohort or comparisons between cohorts).* denotes p<0.05, ** p<0.01, *** p<0.001 by Wilcoxon matched-pairs signed rank test. $ denotes p<0.05, $ $ p<0.01, $ $ $ p<0.001 by Mann-Whitney test.

We first evaluated whether reductions in mycobacterial burden following TB treatment were associated with an increase in polyfunctional PPD-specific CD4 T-cells. The gating scheme used for the cytokine and chemokine analysis is shown in Fig 1A.

We did not observe differences in the total frequency of cytokine/chemokine producing memory CD4 T-cells or in the frequency of IFN-γ, TNF-α, or MIP-1β produced by memory CD4 T-cells following TB therapy in either cohort. We did, however, observe small increases in the frequency of IL-2 production by memory CD4 T-cells in both cohorts (Fig 1B).

When analyzing the co-production of multiple cytokines/chemokines (Fig 1C) by PPD-specific CD4 T-cells we observed a subtle increase in the capacity of PPD-specific CD4 T-cells to simultaneously produce all four cytokines/chemokines tested following TB therapy in the TB cohort (p = 0.008). In both the HIV-TB and TB cohorts we observed higher frequencies of IFN-γ^+^IL-2^+^TNF-α^+^ (p = 0.0004 and p = 0.0004, respectively) and IFN-γ^+^IL-2^+^ (p = 0.0002 and p = 0.02, respectively) subsets following TB treatment. In contrast, the proportion of multiple MIP-1β-producing subsets decreased following TB treatment. For example, PPD-specific CD4 T- cells that were IFN-γ^+^TNF-α^+^MIP-1β^+^ and TNF-α^+^MIP-1β^+^ decreased in our TB cohort (p = 0.03 and p = 0.04, respectively), while PPD-specific CD4 T-cells that were IFN-γ^+^MIP-1β^+^ and MIP-1β^+^-only decreased in our HIV-TB cohort (p = 0.05 and p = 0.006, respectively). Finally, the subset of cells producing TNF-α-only decreased following TB therapy in our TB cohort (p = 0.03).

These data suggest that while reductions in mycobacterial burden through TB treatment may not necessarily increase the frequency of polyfunctional PPD-specific CD4 T-cells, it does alter the functional profile of PPD-specific CD4 T-cells, specifically with respect to IL-2 and MIP-1β producing subsets.

### PPD-specific CD4 T-cells become less differentiated following TB treatment

**Fig 2:**
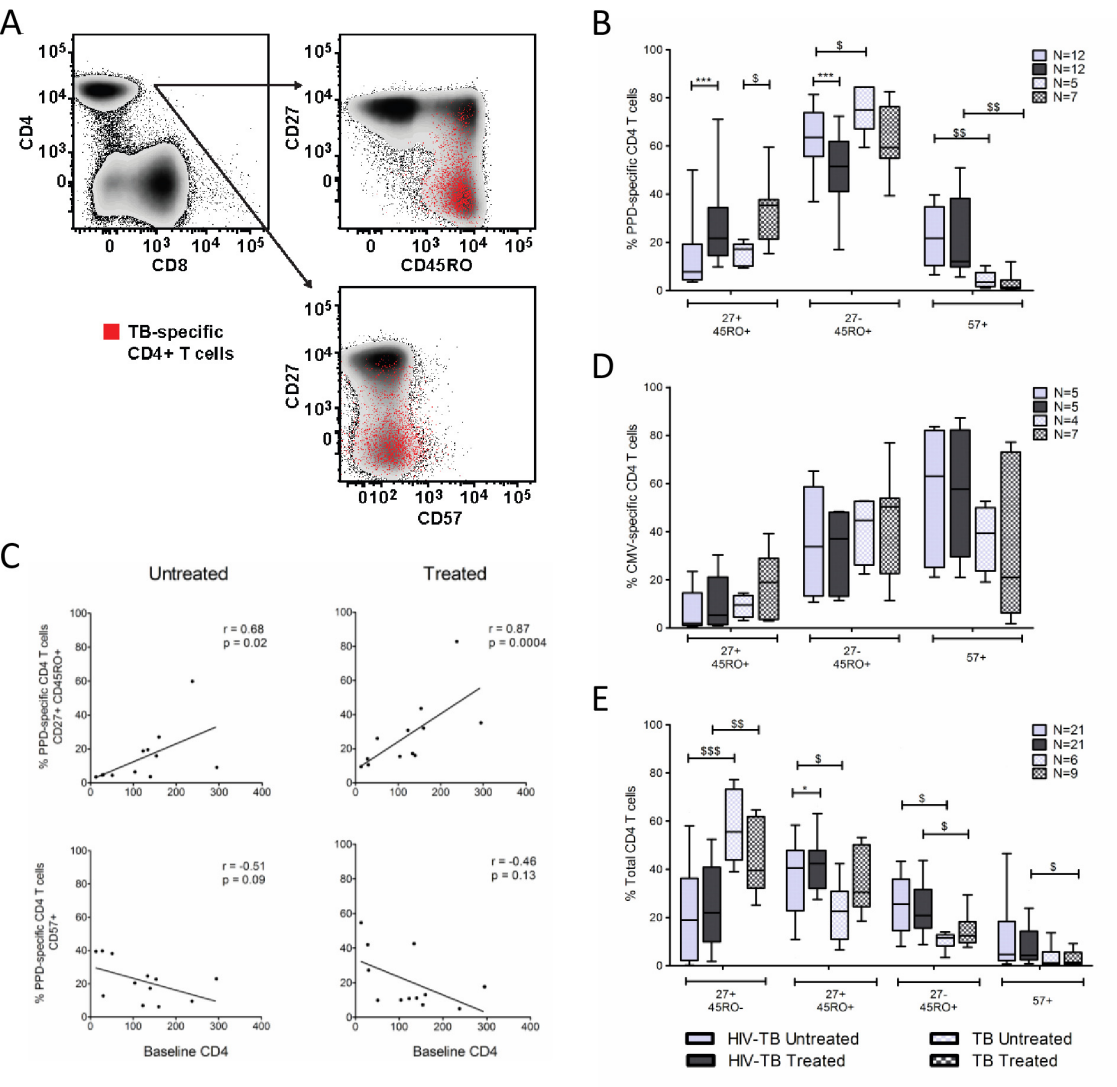
TB therapy alters maturation phenotype of PPD-specific CD4 T-cells. **A.** Representative example of differentiation marker expression on PPD-specific CD4 T-cells. PPD-specific CD4 T-cells (red dots) are overlaid onto density plots of CD27 and CD45RO and CD27 and CD57, gated on total CD4 T-cells. **B.** Frequency of PPD-specific CD4 T-cells expressing CD27^+^CD45RO^+^ (CM), CD27^-^CD45RO^+^ (EM), and CD57^+^ (TD) phenotypes. **C.** Correlation between baseline CD4 T-cell count and frequency of PPD-specific CD4 T-cells expressing CM and TD phenotypes in untreated and treated TB disease. Lines of best fit, along with Spearman’s rank correlation coefficient and corresponding p-values are shown **D.** Frequency of CMV-specific CD4 T-cells expressing CM, EM, and TD phenotypes in our HIV-TB and TB cohorts. **E.** Frequency of naïve, CM, EM, and TD subsets on total CD4 T-cells in untreated and treated TB disease in our HIV-TB and TB cohorts. To assess maturation phenotype on PPD and CMV-specific CD4 T-cells, only samples with at least 50 cytokine positive cells and 2-fold higher responses than negative control samples were included to allow for a statistically valid analysis. The Wilcoxon matched-pairs signed rank test was used for paired comparisons (HIV-TB cohort) while the Mann-Whitney test was used to analyze unpaired data (TB cohort and comparisons between cohorts). * denotes p<0.05, ** p<0.01, *** p<0.001 by Wilcoxon matched-pairs signed rank test. $ denotes p<0.05, $ $ p<0.01, $ $ $ p<0.001 by Mann-Whitney test.

We next evaluated the effects of TB treatment on the maturational profile of PPD-specific CD4 T-cells. We simultaneously measured CD27, CD45RO, and CD57 expression on PPD-specific CD4 T-cells which discriminates between naïve (CD27^+^CD45RO^-^), central memory-like (CD27^+^CD45RO^+^, CM), effector memory-like (CD27^-^CD45RO^+^, EM), and terminally differentiated (CD57^+^, TD) CD4 T-cells (Fig 2A)[26].

PPD-specific CD4 T-cells in untreated TB predominantly exhibited an EM phenotype (Fig 2B). Following TB therapy, the proportion of PPD-specific CD4 T-cells expressing an EM phenotype decreased in our HIV-TB cohort (p = 0.0015), while a trend towards decreased expression was observed in our TB cohort. The proportion of PPD-specific CD4 T-cells expressing a CM phenotype increased in both our HIV-TB and TB cohorts following TB treatment (p = 0.001 and p = 0.02, respectively). Within our HIV-TB cohort, the proportion of PPD-specific CD4 T-cells expressing a CM phenotype correlated directly with baseline CD4 count (Fig 2C). TB treatment did not influence the proportion of TD PPD-specific CD4 T-cells. However, the proportion of TD PPD-specific CD4 T-cells in the TB cohort was lower than that observed in our HIV-TB cohort in both untreated (p = 0.005) and treated (p = 0.003) TB disease.

As additional controls we analyzed the maturational profile of CMV-specific CD4 T-cells and the total CD4 T cell population. Patients included in the CMV analysis were not known to have CMV disease but rather identified based on response to CMV antigen stimulation. No differences were noted in the frequency of CMV-specific CD4 T-cells exhibiting CM, EM, or TD phenotypes following TB treatment in either cohort (Fig 2D). Although there was a subtle increase in the total CD4 T-cell CM compartment following ART and TB therapy in the HIV-TB cohort (median 40.6% vs. 42.3%, p = 0.04), a similar change was not observed in the TB cohort (Fig 2E). Furthermore, we did not appreciate differences in frequency of EM or TD subsets within the total CD4 T-cell population following treatment.

Our data suggest that the altered maturation profile of PPD-specific CD4 T-cells after TB treatment is pathogen-specific and does not represent a generalized effect of TB treatment on CD4 T-cell differentiation.

### PD-1 and CTLA-4 expression on PPD-specific CD4 T-cells decreases following TB therapy

**Fig 3:**
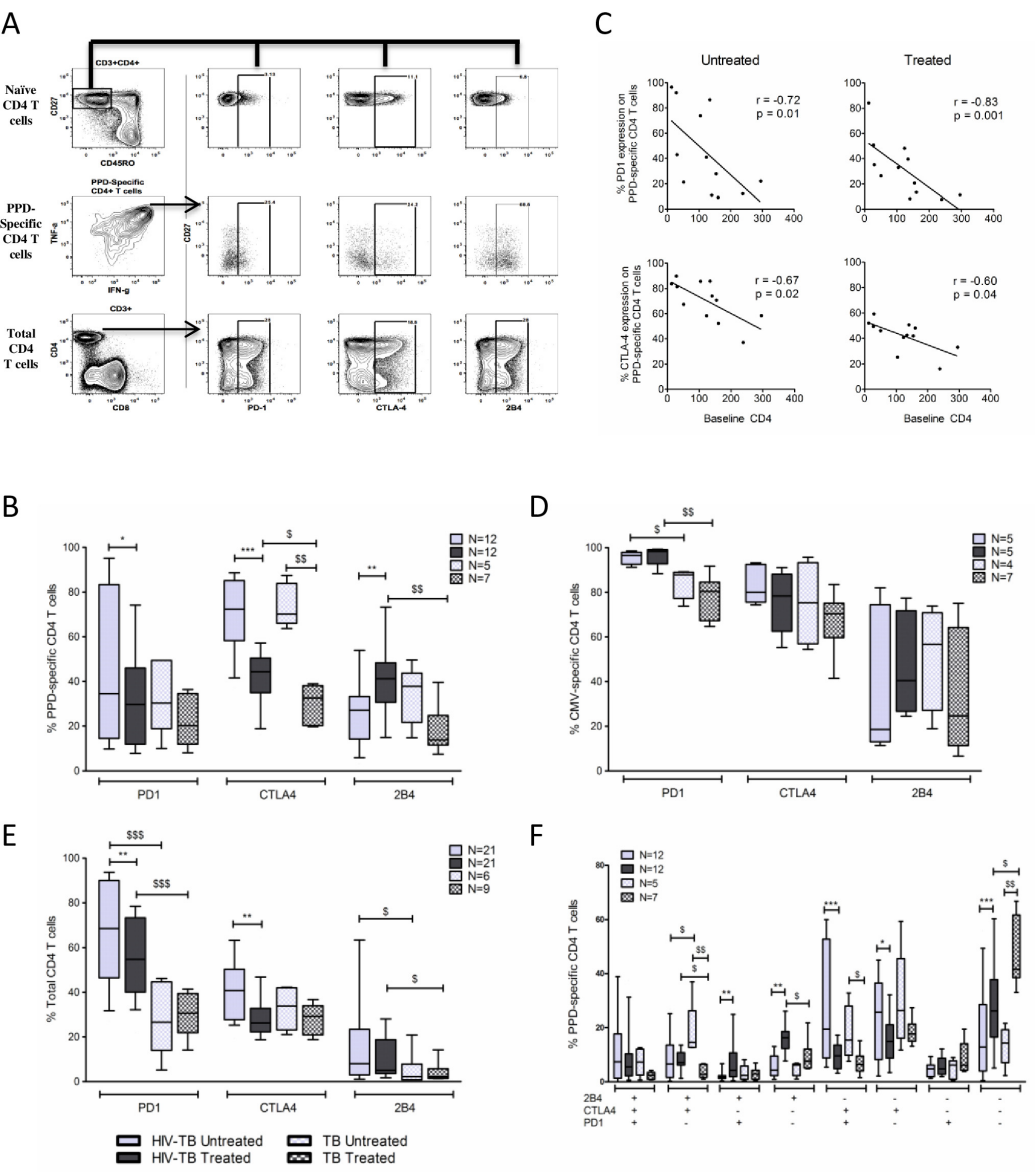
PD-1 and CTLA-4 expression on PPD-specific CD4 T-cells in response to TB treatment. **A.** Representative plot showing the gating scheme used to identify PD-1, CTLA-4, and 2B4 expression on PPD-specific CD4 T-cells. Single, live, CD3^+^, CD4^+^ cells were gated for CD27 and CD45RO. The naïve CD4 T cell population was identified (CD27^+^CD45RO^-^) and selected to set gates for PD-1, CTLA-4 and 2B4 as this population typically does not express inhibitory molecules. These gates were then applied to PPD-specific and total CD4 T-cell populations. **B.** Expression of PD-1, CTLA-4, and 2B4 on PPD-specific CD4 T-cells in untreated and treated TB disease. **C.** Correlation between baseline CD4 T-cell count and frequency of PD-1 and CTLA-4 expression on PPD-specific CD4 T-cells in untreated and treated TB disease. Lines of best fit, along with Spearman’s rank correlation coefficient and corresponding p-values are shown **D.** Expression of PD-1, CTLA-4, and 2B4 on CMV-specific CD4 T-cells in untreated and treated TB disease in our HIV-TB and TB cohorts. **E**. Expression of PD-1, CTLA-4, and 2B4 on total CD4 T-cells in untreated and treated TB disease in our HIV-TB and TB cohorts. **F**. Bar graph depicts the co-expression patterns of PD-1, CTLA-4, and 2B4 on PPD-specific CD4 T-cells in untreated and treated TB disease in our HIV-TB and TB cohorts. To assess expression of inhibitory molecules on PPD and CMV-specific CD4 T-cells, only samples with at least 50 cytokine positive cells and 2-fold higher responses than negative control samples were included to allow for a statistically valid analysis. * denotes p<0.05, ** p<0.01, *** p<0.001 by Wilcoxon matched-pairs signed rank test. $ denotes p<0.05, $ $ p<0.01, $ $ $ p<0.001 by Mann-Whitney test.

We evaluated PD-1, CTLA-4, and 2B4 (CD244) expression on PPD-specific CD4 T-cells in both untreated and treated TB disease. The gating strategy is depicted in Fig 3A and crude data from a representative donor within each cohort is shown in S2 Fig.

In our HIV-TB cohort, most PPD-specific CD4 T-cells in untreated TB disease expressed CTLA-4 (median 72.3%, Fig 3B). Concurrent ART and TB therapy reduced CTLA-4 expression by approximately 40% (p = 0.0005). PD-1 expression on PPD-specific CD4 T-cells was highly variable in untreated TB but decreased with concurrent ART and TB therapy (p = 0.03). Both PD-1 and CTLA-4 expression were inversely correlated with baseline CD4 count (Fig 3C). Within our TB cohort, TB therapy reduced CTLA-4 expression on PPD-specific CD4 T-cells by 60% (p = 0.0025) while only a trend towards reduced PD-1 expression was observed (33.4% vs. 20.9%, p = 0.25, Fig 3B).

PD-1 and CTLA-4 expression on CMV-specific CD4 T-cells (Fig 3D) and total CD4 T-cells (Fig 3E) was evaluated as a control. In both cohorts expression of PD-1 and CTLA-4 on CMV-specific CD4 T-cells was high in untreated TB disease and remained unchanged following ART and TB therapy. PD-1 expression on total CD4 T-cells was higher in the HIV-TB cohort compared to the TB cohort and concurrent ART and TB treatment reduced PD-1 expression on total CD4 T-cells. However, no change in PD-1 expression on total CD4 T-cells was noted in our TB cohort following TB treatment. CTLA-4 expression on total CD4 T-cells was similar between cohorts and lower than that observed on PPD-specific CD4 T-cells. Concurrent ART and TB therapy reduced CTLA-4 expression on total CD4 T-cells in our HIV-TB cohort but no change was observed in our TB cohort following TB treatment. Taken together, these data imply that observed decreases in PD-1 and CTLA-4 expression on PPD-specific CD4 T-cells are related to TB treatment, not ART, are pathogen-specific and does not represent generalized effects of TB therapy on total CD4 T-cells.

2B4 (CD244) expression on PPD-specific CD4 T-cells was modest in untreated TB disease. While TB treatment increased expression levels in the HIV-TB cohort, the proportion of PPD-specific CD4 T-cells expressing 2B4 declined in the TB cohort (Fig 3B).

Finally, we analyzed co-expression patterns of these molecules on PPD-specific CD4 T-cells (Fig 3F). In untreated TB disease most PPD-specific CD4 T-cells expressed either CTLA-4 alone or in combination with PD-1. TB treatment reduced the frequencies of these specific subsets while increasing the frequency of PPD-specific CD4 T-cells that were PD-1^-^CTLA-4^-^2B4^-^ (p = 0.0005 and p = 0.0025 for HIV-TB and TB cohorts, respectively).

## Discussion

We simultaneously characterized the cytokine profile, differentiation state, and inhibitory molecule expression on PPD-specific CD4 T-cells, as a surrogate for Mtb-specific CD4 T-cell response, in individuals undergoing treatment for active TB disease. In our longitudinal analysis of HIV-TB co-infected individuals, we found that TB treatment (1) altered the functional profile of PPD-specific CD4 T-cells, specifically subsets producing MIP-1β and IL-2, (2) reduced the frequency of PPD-specific CD4 T-cells expressing an EM phenotype while simultaneously increasing the proportion with a CM phenotype, and (3) reduced expression of PD-1 and CTLA-4 on PPD-specific CD4 T-cells. A cross-sectional analysis of TB mono-infected patients with untreated and treated TB disease revealed similar patterns of expression suggesting these changes are related to anti-tuberculous therapy rather than co-administration of ART.

Several groups have recently demonstrated that latent TB infection and AFB sputum smear negative active TB disease are characterized by higher frequencies of Mtb-specific CD4 T-cells co-producing IFN-γ/IL-2/TNF-α compared to AFB sputum smear positive active TB disease [13, 14]. Following treatment for TB disease, we found higher frequencies of PPD-specific CD4 T-cells that were IFN-γ^+^IL-2^+^TNF-α^+^ in both our cohorts. This adds to the existing literature correlating lower *M*. *tuberculosis* burden with higher frequencies of Mtb-specific CD4 T-cells co-producing IFN-γ/IL-2/TNF-α.

We also observed a higher frequency of PPD-specific CD4 T-cell subsets producing MIP-1β in untreated TB disease when compared to treated TB disease. The capacity of pathogen-specific CD4 T-cells to produce IL-2 diminishes, while the capacity to produce MIP-1β increases as cells become more differentiated [27]. The higher proportion of CM and lower proportion of EM PPD-specific CD4 T-cells following TB treatment could account for these differences. Another explanation could be the reversal of T-cell exhaustion following TB treatment. Exhausted T-cells typically lose function in a hierarchical manner where reduced IL-2 production and proliferative capacity are lost first [17]. TB treatment lowered expression of PD-1 and CTLA-4 on PPD-specific CD4 T-cells which could have reversed T-cell dysfunction, increasing IL-2 production by PPD-specific CD4 T-cells.

The shifts in PPD-specific CD4 T-cell differentiation following TB treatment reflect changes in CD27 expression. Differences in CD27 expression on Mtb-specific CD4 T-cells in active and latent TB infection have been previously reported [28, 29]. CD27 is a co-stimulatory receptor that is downregulated as CD4 T-cells proceed from an early (central memory) to a late differentiated stage (effector memory). The degree to which T-cells differentiate is in part dictated by the magnitude and duration of antigenic exposure [30]. The increased mycobacterial burden and prolonged antigen exposure during active TB disease drives differentiation of Mtb-specific CD4 T cells towards an EM phenotype. Although TB treatment reduced mycobacterial burden and CD27 expression on PPD-specific CD4 T-cells increased with treatment, the majority of these cells continued to express an EM phenotype following treatment. While this could reflect delayed re-expression of CD27, a more likely explanation is that this reflects reduced differentiation from naïve to CM antigen-specific CD4 T-cells during late stages of treatment, a period of low antigen burden. This is supported by data showing CD27^lo^ T-cells are more likely to undergo apoptosis than regain CD27 expression [31].

Both CTLA-4 and PD-1 expression on PPD-specific CD4 T-cells decreased with TB treatment, though the effect was greater for CTLA-4. Temporal differences in CTLA-4 and PD-1 expression could account for this. CTLA-4 is thought to play a dominant role in limiting T-cell activity early in the immune response, and may therefore be downregulated ahead of PD-1 [32]. The decreased expression was observed in both our HIV-TB and TB cohorts and was not observed on CMV-specific CD4 T-cells suggesting the reduced expression is pathogen-specific, related to decreased mycobacterial burden, and less likely driven by co-administration of ART. The high frequency of PD-1 expression on CMV-specific, compared to PPD-specific, CD4 T-cells may represent an inherent characteristic of the pathogen-specific response while also reflecting an exhausted phenotype that is typically seen in antigen-specific T-cells during states of persistent antigen exposure and is in line with previously published data on viral-specific CD4 T-cells [21]. We did not find consistent patterns of 2B4 expression on PPD-specific CD4 T-cells in response to TB treatment. Little is known about 2B4 expression in TB infection. In a recent publication, 2B4 expression was higher on TB-specific T-cells in active disease than in latent infection, however, expression increased on TB-specific T-cells with longer duration of treatment [33].

In chronic viral infections the progressive accumulation of multiple inhibitory molecules on T-cells can lead to T-cell dysfunction and poor viral control [34–36]. In both cohorts the majority of PPD-specific CD4 T-cells either expressed CTLA-4 alone or co-expressed PD-1 and CTLA-4 in untreated TB disease. The frequency of these specific subsets decreased following treatment while the proportion of PPD-specific CD4 T-cells that was negative for all three inhibitory molecules increased with treatment. Our findings extend observations made in the setting of chronic viral infections to TB infection, however, the significance of these findings is uncertain. Increased expression of these molecules in untreated TB disease could simply represent a state of increased T-cell activation or it may be that increased expression and subsequent T-cell inhibition could trigger disease progression. In pulmonary TB, blockade of the PD-1 pathway enhances IFN-γ production by lymphocytes, rescues TB-specific T-cells from apoptosis, and increases their proliferative capacity [24, 37]. This suggests that PD-1 mediated T-cell inhibition could dampen the protective T-cell response during tuberculosis infection. However, PD-1 knockout mice have demonstrated reduced survival and increased bacterial burden compared to their wild-type counterparts suggesting PD-1 may play a protective role by limiting immunopathology in TB disease [38, 39]. Similarly, while CTLA-4 blockade in mice infected with *M*. *bovis* enhanced lymphocyte proliferation and IFN-γ production, no effect on mycobacterial clearance was observed [40]. Therefore, PD-1 and CTLA-4 may play a homeostatic role in controlling the excessive inflammation that occurs in TB disease, but overexpression on T-cells during latent infection could inhibit protective T-cell responses leading to disease progression.

Although we characterized longitudinally the phenotype and function of Mtb-specific CD4 T-cells in 21 individuals undergoing treatment for HIV-TB co-infection and replicated our findings in a second cohort of HIV negative individuals, both cohorts were modest in size. Differences in the functional profile of Mtb-specific CD4 T-cells have been observed in individuals with smear positive and smear negative TB [14]. Unfortunately, quantitative AFB smear grades were not available for all individuals in our cohorts limiting our ability to investigate this association. The co-administration of ART (early vs. deferred) to individuals in the HIV-TB cohort along with HIV infection by itself can make it difficult to discern effects attributable to TB treatment alone. However, all 21 individuals in our HIV-TB cohort suppressed their viral loads by week 48 and we did not observe differences in the functional profile, maturation phenotype or expression of inhibitory molecules between study arms (S3 Fig). More importantly, we observed similar patterns of cytokine/chemokine expression, maturation profile, and inhibitory molecule expression on Mtb-specific CD4 T-cells following TB treatment in our TB cohort. This supports our hypothesis that TB treatment-related reductions in mycobacterial burden largely influence the phenotype and function of Mtb-specific CD4 T-cells. Finally, while we included individuals from A5221 who received TB therapy for up to one month, the majority (16/21) of patients enrolled in our cohort received TB therapy for less than 14 days prior to inclusion. The impact of this short duration of treatment on Mtb-specific CD4 T-cell responses is unclear, but we believe any impact would likely underestimate differences between the untreated and treated groups.

In sum, we have identified several functional and phenotypic attributes of Mtb-specific CD4 T-cells that are altered with TB treatment in individuals with and without HIV infection. Our study shows that the phenotype, function, and inhibitory molecule expression on Mtb-specific CD4 T-cells can provide valuable immunological information about mycobacterial burden. These data provide insights into potential new ways of monitoring response to therapy and provide support for future studies evaluating whether the function, inhibitory molecule expression, or maturation phenotype of Mtb-specific CD4 T-cells can predict development of clinical TB disease.

## Supporting Information

S1 Fig PPD-specific CD4 T-cell gating strategy. PBMCs stimulated with PPD were analyzed using flow cytometry. We used SSC-A and FSC–A to remove debris and identify our small lymphocyte population. Next, single cell populations were identified by applying FSC-H vs FSC-A gates. Dead cells were eliminated using Aqua live/dead stain. T cells were identified using a CD3 gate. A CD4 vs. CD8 gate was applied to the total CD3^+^ population to identify CD4^+^ T-cells. CD27 and CD45RO gates were then applied to identify the total memory CD4 T-cell population. Cytokine/chemokine expression within the total memory CD4 T-cell compartment was assessed and total memory CD4 T-cells producing IFN-γ, IL-2, TNF-α, or MIP-1β were classified as PPD-specific CD4 T-cells.
(TIF)

S2 Fig Inhibitory molecule expression on PPD-specific and total CD4 T-cells in representative donors from the HIV-TB and TB cohorts. **A.** Histograms depicting PD-1, CTLA-4 and 2B4 expression on PPD-specific and total CD4 T-cells in untreated (red line) and treated (blue line) TB disease in a HIV-TB co-infected individual receiving concurrent ART and TB treatment. **B.** Histogram depicting PD-1, CTLA-4, and 2B4 expression on PPD-specific and total CD4 T-cells in a HIV negative individual with untreated TB disease (red line) and a HIV negative individual with treated TB disease (blue line).
(TIF)

S3 Fig Functional profile, maturation phenotype, and inhibitory molecule expression on PPD-specific CD4 T-cells are not impacted by early versus deferred administration of ART. **A.** Cytokine/chemokine profile of PPD-specific CD4 T-cells at week 48 in the HIV-TB cohort based on treatment group assignment, early (light gray bar) vs. deferred ART (dark gray bar). **B.** Frequency of PPD-specific CD4 T-cells expressing CD27^+^CD45RO^+^ (CM), CD27^-^CD45RO^+^ (EM), and CD57^+^ (TD) phenotypes at week 48 in the HIV-TB cohort based on treatment group assignment. **C.** Expression of PD-1, CTLA-4, and 2B4 on TB-specific CD4 T-cells at week 48 in the HIV-TB cohort based on treatment group assignment. For all bar graphs, bars denote IQR, horizontal lines denote median, whiskers denote 10^th^ to 90^th^ percentiles. The Mann-Whitney test was used to analyze differences between groups. $ denotes p<0.05, $ $ p<0.01, $ $ $ p<0.001.
(TIF)
