# Supplementary figures and images for: Tuberculosis Therapy Modifies the Cytokine Profile, Maturation State, and Expression of Inhibitory Molecules on Mycobacterium tuberculosis-Specific CD4+ T-Cells
Source: PLoS One. 2016 Jul 1;11(7):e0158262. doi: 10.1371/journal.pone.0158262 (PMC4930205; doi:10.1371/journal.pone.0158262)

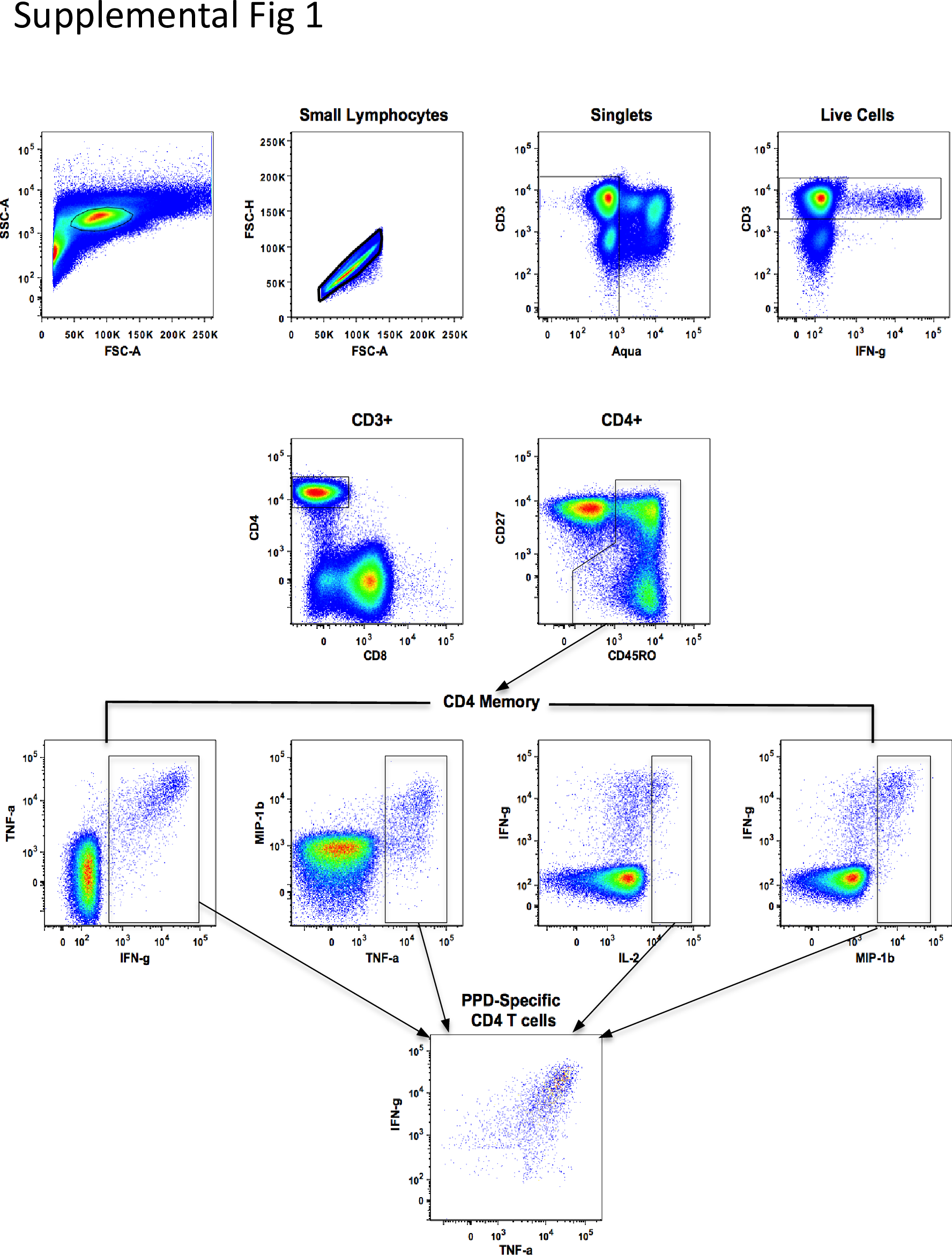

Supplement: S1 Fig — PBMCs stimulated with PPD were analyzed using flow cytometry. We used SSC-A and FSC–A to remove debris and identify our small lymphocyte population. Next, single cell populations were identified by applying FSC-H vs FSC-A gates. Dead cells were eliminated using Aqua live/dead stain. T cells were identified using a CD3 gate. A CD4 vs. CD8 gate was applied to the total CD3+ population to identify CD4+ T-cells. CD27 and CD45RO gates were then applied to identify the total memory CD4 T-cell population. Cytokine/chemokine expression within the total memory CD4 T-cell compartment was assessed and total memory CD4 T-cells producing IFN-γ, IL-2, TNF-α, or MIP-1β were classified as PPD-specific CD4 T-cells. (TIF) [file pone.0158262.s001.tif]

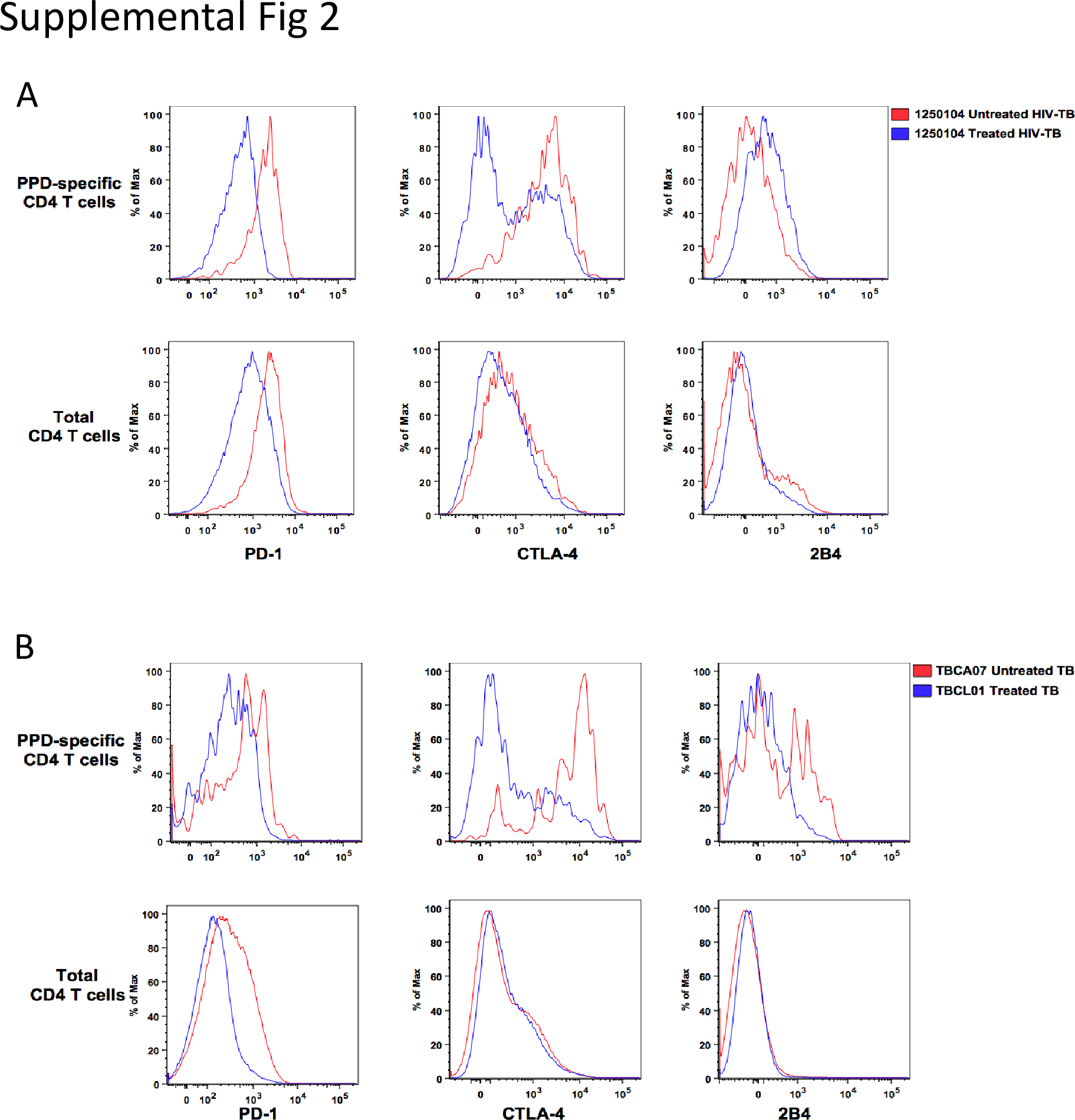

Supplement: S2 Fig — A. Histograms depicting PD-1, CTLA-4 and 2B4 expression on PPD-specific and total CD4 T-cells in untreated (red line) and treated (blue line) TB disease in a HIV-TB co-infected individual receiving concurrent ART and TB treatment. B. Histogram depicting PD-1, CTLA-4, and 2B4 expression on PPD-specific and total CD4 T-cells in a HIV negative individual with untreated TB disease (red line) and a HIV negative individual with treated TB disease (blue line). (TIF) [file pone.0158262.s002.tif]

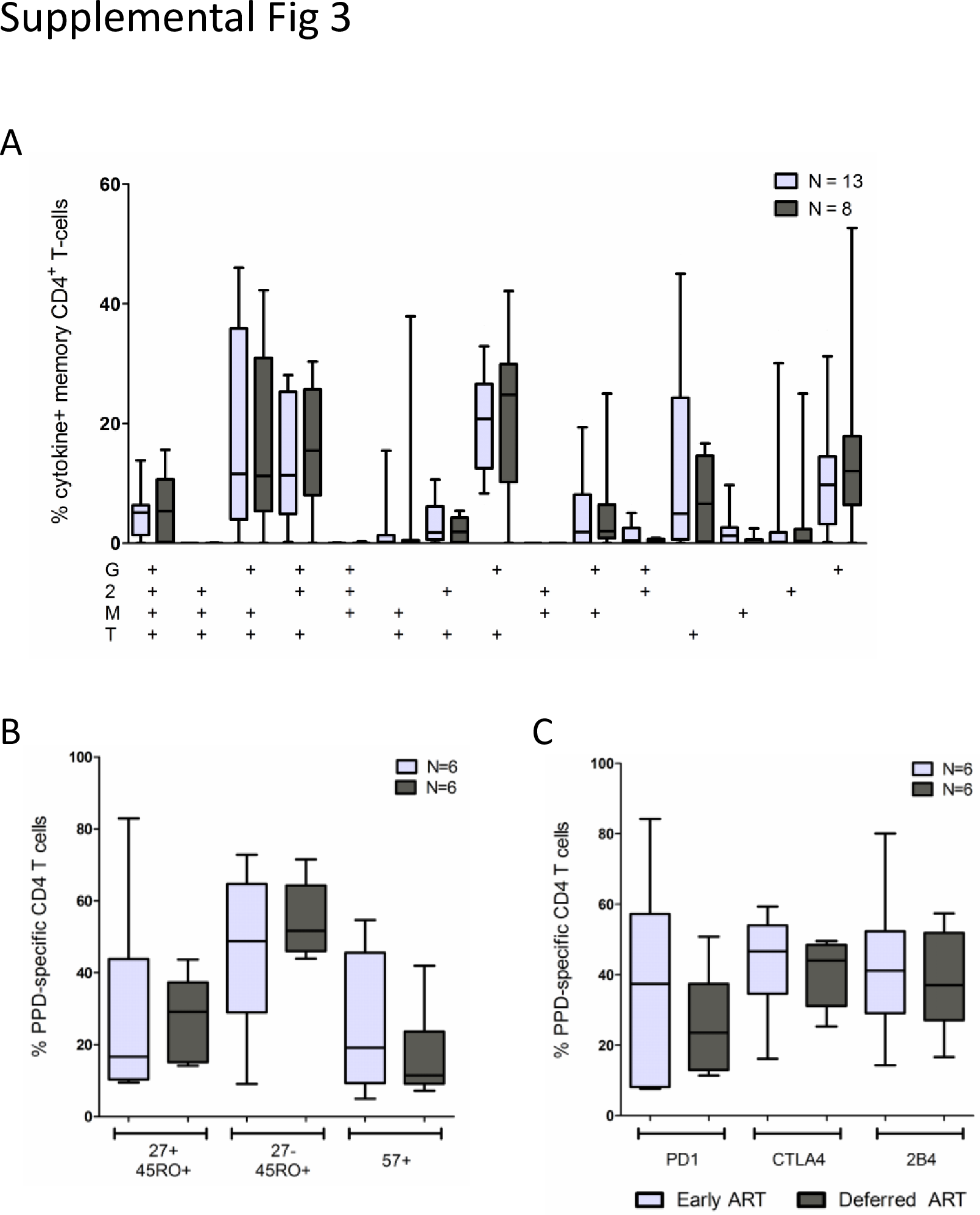

Supplement: S3 Fig — A. Cytokine/chemokine profile of PPD-specific CD4 T-cells at week 48 in the HIV-TB cohort based on treatment group assignment, early (light gray bar) vs. deferred ART (dark gray bar). B. Frequency of PPD-specific CD4 T-cells expressing CD27+CD45RO+ (CM), CD27-CD45RO+ (EM), and CD57+ (TD) phenotypes at week 48 in the HIV-TB cohort based on treatment group assignment. C. Expression of PD-1, CTLA-4, and 2B4 on TB-specific CD4 T-cells at week 48 in the HIV-TB cohort based on treatment group assignment. For all bar graphs, bars denote IQR, horizontal lines denote median, whiskers denote 10th to 90th percentiles. The Mann-Whitney test was used to analyze differences between groups. $ denotes p<0.05, $ $ p<0.01, $ $ $ p<0.001. (TIF) [file pone.0158262.s003.tif]
